# Supplementary material for: From sequence to enzyme mechanism using multi-label machine learning
Source: BMC Bioinformatics. 2014 May 19;15:150. doi: 10.1186/1471-2105-15-150 (PMC4229970; doi:10.1186/1471-2105-15-150)
Supplement: Additional file 2 — Java code of ml2db. Additional file ml2db_code.tar.gz contains the Java source code to run the multi-label machine learning experiments and save the results to database. The code’s Javadoc is included. [file 1471-2105-15-150-S2.zip › additional file 2/ml2db/ml2db/doc/overview-summary.html]

Overview


JavaScript is disabled on your browser.


- Overview
- Package
- Class
- Use
- Tree
- Deprecated
- Index
- Help

- Prev
- Next

- Frames
- No Frames

- All Classes

Packages

| Package | Description |
| --- | --- |
|  |  |
| --- | --- |
| main.ml2db |  |
| main.ml2db.al |  |
| main.ml2db.al.database |  |
| main.ml2db.al.learning |  |
| main.ml2db.al.utility |  |
| main.ml2db.al.utility.confidence |  |
| main.ml2db.al.utility.globaldistance |  |
| main.ml2db.al.utility.hybrid |  |
| main.ml2db.al.utility.neighbours |  |
| main.ml2db.al.utility.pickers |  |
| main.ml2db.al.utility.pickers.byfrequency |  |
| main.ml2db.graphviz |  |
| main.ml2db.mulan.attributesfiltered |  |
| main.ml2db.mulan.classifier.lazy |  |
| main.ml2db.mulan.learn |  |
| main.ml2db.mulan.learn.crosseval |  |
| main.ml2db.mulan.learn.evaluation |  |
| main.ml2db.mulan.learn.traintest |  |
| main.ml2db.mulan.measuremetrics |  |
| main.ml2db.mulan.mulanarff |  |
| main.ml2db.mulan.mulanarff.database |  |
| main.ml2db.mulan.predict |  |
| main.ml2db.weka |  |
| test.ml2db |  |
| test.ml2db.al |  |
| test.ml2db.al.utility.confidence |  |
| test.ml2db.al.utility.globaldistance |  |
| test.ml2db.al.utility.hybrid |  |
| test.ml2db.al.utility.neighbourhood |  |
| test.ml2db.al.utility.pickers |  |
| test.ml2db.alltests |  |
| test.ml2db.dataharness |  |
| test.ml2db.graphviz |  |
| test.ml2db.mulan.attributesfilter |  |
| test.ml2db.mulan.classifier.lazy |  |
| test.ml2db.mulan.learn |  |
| test.ml2db.mulan.learn.database |  |
| test.ml2db.mulan.learn.traintest |  |
| test.ml2db.mulan.measuremetrics |  |
| test.ml2db.mulan.mulanarff |  |
| test.ml2db.mulan.mulanarff.database |  |
| test.ml2db.mulan.predict |  |
| test.ml2db.weka |  |

- Overview
- Package
- Class
- Use
- Tree
- Deprecated
- Index
- Help

- Prev
- Next

- Frames
- No Frames

- All Classes
